# Supplementary material for: Causal Relationship between Aging and Anorexia Nervosa: A White-Matter-Microstructure-Mediated Mendelian Randomization Analysis
Source: Biomedicines. 2024 Aug 16;12(8):1874. doi: 10.3390/biomedicines12081874 (PMC11351342; doi:10.3390/biomedicines12081874)
Supplement: Supplementary file 1 [file biomedicines-12-01874-s001.zip › Supplementary Figure/Figure S4/Singe SNP plot of MR analysis for TL to ubm-b-1547.pdf]

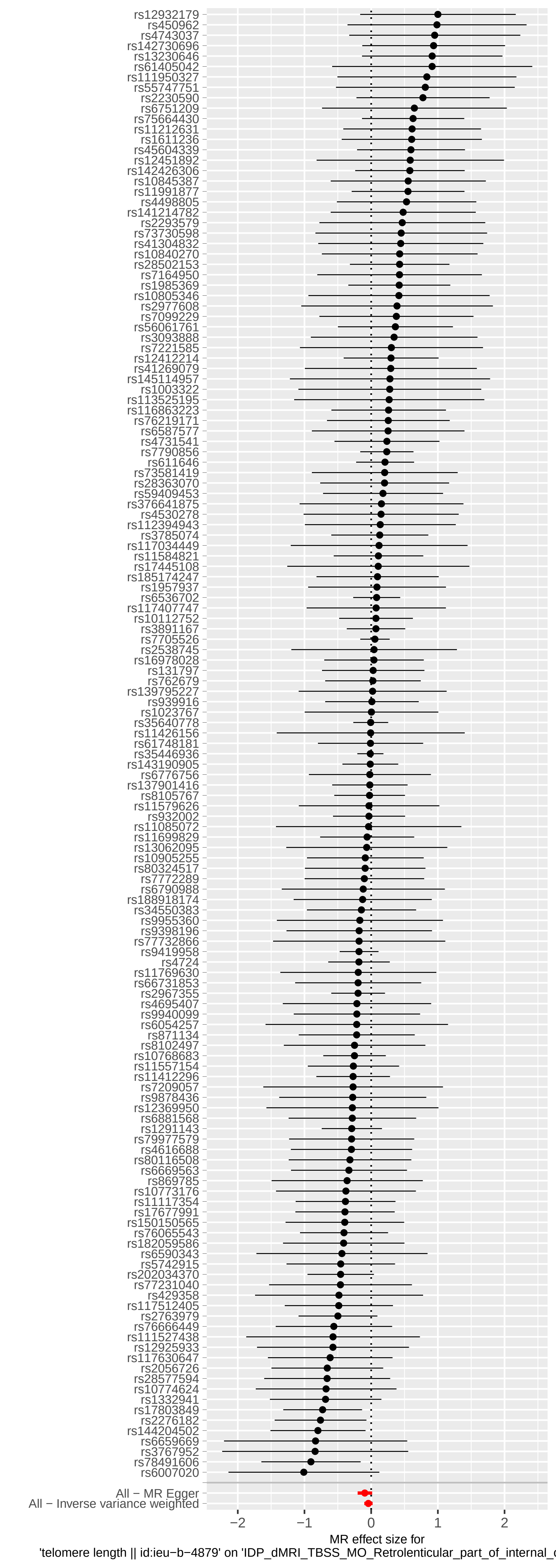
rs12932179
rs450962
rs4743037
rs142730696
rs13230646
rs61405042
rs111950327
rs55747751
rs2230590
rs6751209
rs75664430
rs11212631
rs1611236
rs45604339
rs12451892
rs142426306
rs10845387
rs11991877
rs4498805
rs141214782
rs2293579
rs73730598
rs41304832
rs10840270
rs28502153
rs7164950
rs1985369
rs10805346
rs2977608
rs7099229
rs56061761
rs3093888
rs7221585
rs12412214
rs41269079
rs145114957
rs1003322
rs113525195
rs116863223
rs76219171
rs6587577
rs4731541
rs7790856
rs611646
rs73581419
rs28363070
rs59409453
rs376641875
rs4530278
rs112394943
rs3785074
rs117034449
rs11584821
rs17445108
rs185174247
rs1957937
rs6536702
rs117407747
rs10112752
rs3891167
rs7705526
rs2538745
rs16978028
rs131797
rs762679
rs139795227
rs939916
rs1023767
rs35640778
rs11426156
rs61748181
rs35446936
rs143190905
rs6776756
rs137901416
rs8105767
rs11579626
rs932002
rs11085072
rs11699829
rs13062095
rs10905255
rs80324517
rs7772289
rs6790988
rs188918174
rs34550383
rs9955360
rs9398196
rs77732866
rs9419958
rs4724
rs11769630
rs66731853
rs2967355
rs4695407
rs9940099
rs6054257
rs871134
rs8102497
rs10768683
rs11557154
rs11412296
rs7209057
rs9878436
rs12369950
rs6881568
rs1291143
rs79977579
rs4616688
rs80116508
rs6669563
rs869785
rs10773176
rs11117354
rs17677991
rs150150565
rs76065543
rs182059586
rs6590343
rs5742915
rs202034370
rs77231040
rs429358
rs117512405
rs2763979
rs76666449
rs111527438
rs12925933
rs117630647
rs2056726
rs28577594
rs10774624
rs1332941
rs17803849
rs2276182
rs144204502
rs6659669
rs3767952
rs78491606
rs6007020
All − MR Egger
All − Inverse variance weighted
−2
−1
0
1
2
MR effect size for
'telomere length || id:ieu-b-4879' on 'IDP_dMRI_TBSS_MO_Retrolenticular_part_of_internal_c
